# Faecal Metabolome Profiles in Individuals Diagnosed with Hyperplastic Polyps and Conventional Adenomas

**DOI:** 10.3390/ijms252413324

**Published:** 2024-12-12

**Authors:** Alberto Valdés, Sergio Ruiz-Saavedra, Nuria Salazar, Alejandro Cifuentes, Adolfo Suárez, Ylenia Díaz, Carmen González del Rey, Sonia González, Clara G. de los Reyes-Gavilán

**Affiliations:** 1 Foodomics Laboratory, Instituto de Investigación en Ciencias de la Alimentación (CIAL), Consejo Superior de Investigaciones Científicas-Universidad Autónoma de Madrid (CSIC-UAM), 28049 Madrid, Spain; a.valdes@csic.es (A.V.); a.cifuentes@csic.es (A.C.); 2 Department of Microbiology and Biochemistry of Dairy Products, Instituto de Productos Lácteos de Asturias, Consejo Superior de Investigaciones Científicas (IPLA-CSIC), 33011 Oviedo, Spain; sergio.ruiz@ipla.csic.es (S.R.-S.); nuriasg@ipla.csic.es (N.S.); 3 Diet, Microbiota and Health Group, Instituto de Investigación Sanitaria del Principado de Asturias (DIMISA-ISPA), 33011 Oviedo, Spain; adolfo.suarez@sespa.es; 4 Digestive Service, Central University Hospital of Asturias (HUCA), 33011 Oviedo, Spain; 5 Digestive Service, Carmen and Severo Ochoa Hospital, 33819 Cangas del Narcea, Spain; yleniads@hotmail.com; 6 Department of Anatomical Pathology, Central University Hospital of Asturias (HUCA), 33011 Oviedo, Spain; carmenchugonzalezdelrey@gmail.com; 7 Department of Functional Biology, University of Oviedo, 33006 Oviedo, Spain

**Keywords:** faecal metabolome, intestinal mucosa, hyperplastic polyps, conventional adenomas, colorectal cancer

## Abstract

Colorectal cancer (CRC) development is a gradual process in which progressive histological alterations of the intestinal mucosa damage occur over years. This process can be influenced by modifiable external factors such as lifestyle and diet. Most CRC cases (>80%) originate from conventional adenomas through the adenomatous pathway and usually harbour dysplastic cells, whereas the serrated pathway is less frequent (<20% cases) and comprises hyperplastic polyps and other polyps containing dysplastic cells. The aim of the present work was to shed light on alterations of the faecal metabolome associated with hyperplastic polyps and conventional adenomas. Metabolites were analysed by Reversed-Phase High-Performance Liquid Chromatography-Quadrupole-Time of Flight Mass Spectrometry (RP/HPLC-Q/TOF-MS/MS) and Hydrophilic Interaction Liquid Chromatography–Quadrupole-Time of Flight Mass Spectrometry (HILIC-Q/TOF-MS/MS) and the results were integrated. Comparisons were performed between controls without mucosal lesions and the polyps’ group, hyperplastic polyps versus conventional adenomas, and hyperplastic polyps or conventional adenomas versus controls. Alterations of metabolites in specific biochemical modules differentiated hyperplastic polyps and conventional adenomas. The metabolome of the hyperplastic polyps was characterized by an enrichment in glycerophospholipids and an altered metabolism of the degradation pathways of xanthines/purines and pyrimidines, whereas the enrichment in some phenolic compounds and disaccharides, all of them from exogenous origin, was the main differential faecal signature of conventional adenomas. Further research could help to elucidate the contribution of diet and the intestinal microbiota to these metabolomics alterations.

## 1. Introduction

Cancer incidence and its associated morbidity and mortality represent a worldwide health concern issue. The progression of colorectal cancer (CRC), which was the third most diagnosed cancer in 2020 and the second in terms of deaths, seems to be strongly linked to modifiable external factors, such as lifestyle and diet [1,2,3,4]. The development of CRC is a gradual process in which progressive intestinal mucosa damage occurs over years [5,6]. Endoscopic detection of polypoid lesions, accompanied by further histological examination of resected polyps or intestinal mucosa samples during colonoscopy, is used in routine clinical practice to determine the degree of histological mucosa alterations and the risk of CRC. Most CRC cases (>80%) originate from conventional adenomas, usually harbouring dysplastic cells, that are formed across the adenoma–carcinoma sequence through phenotypic changes occurring due to typical genetic alterations from the adenomatous pathway of carcinogenesis. The alternative serrated pathway of carcinogenesis is less frequent (<20%) and is linked to the progression of serrated lesions to carcinoma. The serrated lesions include hyperplastic polyps, usually harbouring hyperplastic cells, as well as traditional serrated adenomas and sessile serrated lesions that normally contain dysplastic cells [7]. Hyperplastic polyps usually present a low risk of evolving to neoplasia, whereas the risk of conventional adenomas is higher [8,9,10].

The faecal metabolome can reflect the intestinal metabolism and health status. It is influenced by the intrinsic metabolism of the host, by food components, and by the intestinal microbiota. Some dietary components that reach the colon undigested, such as some complex carbohydrates (fibres), polyphenols, and, to a more reduced extent, peptides and fats, can be transformed into derived compounds by a wide range of microorganisms from the colonic microbiota, contributing to their assimilation or elimination by faeces [11,12].

Traditionally, most metabolomics studies have focused on CRC and the comparison with healthy controls, whereas less information was available regarding metabolome alterations at initial stages of the intestinal mucosal lesions before the onset of CRC [13,14,15,16]. However, some recent studies have focused on the identification of serum and faecal metabolites during the adenoma–carcinoma progression in order to identify early biomarkers that could improve diagnosis and treatment [17,18,19,20,21]. Several faecal metabolites and specific changes in the serum and faecal metabolome have been found to be associated with the progression of intestinal mucosa lesions within the adenoma–carcinoma sequence [13,14,15,16,17,18,19,20,21]. In addition, some dietary components and/or their combinations (such as dietary fibre, polyphenols, zinc, selenium, some vitamins, among other) have been found to be protective against CRC, whereas the consumption of others (industrial trans-fatty acids, monosodium glutamate, high-fructose corn syrup, etc.) can increase the risk of this type of cancer [22]. Despite this, the metabolomics studies on the serrated pathway of carcinogenesis continue to be still limited. 

**Figure 1:**
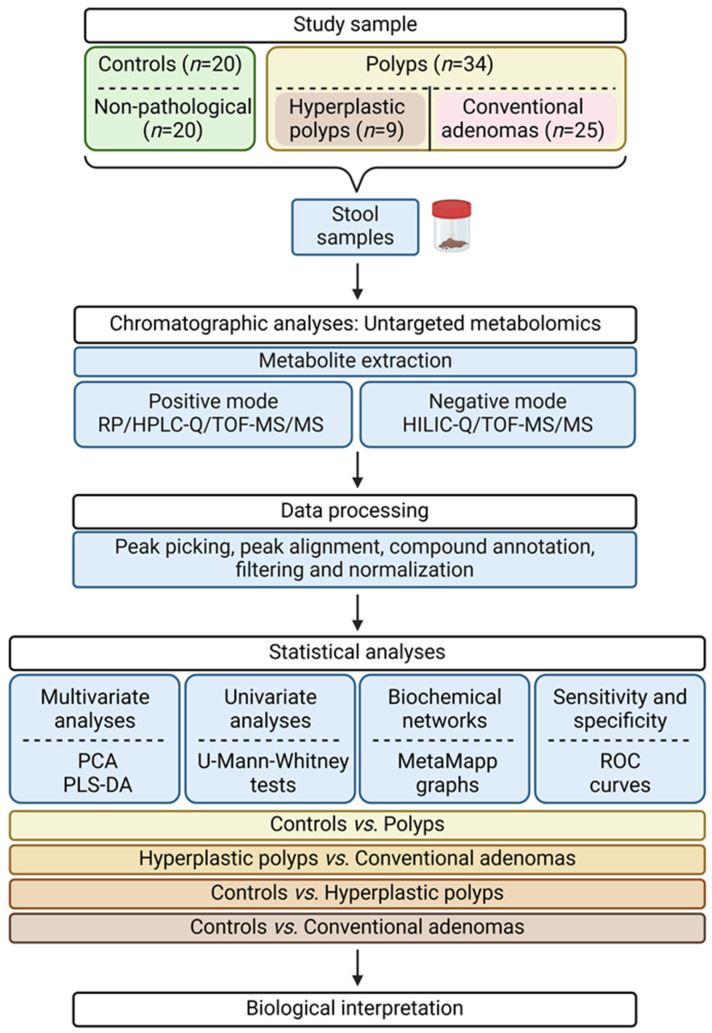
Flowchart of this study. Faecal samples from individuals without intestinal mucosa lesions (non-pathological) and individuals diagnosed with hyperplastic polyps and conventional adenomas were collected and subjected to untargeted metabolomics. Subsequent analysis identified discriminant metabolites differentiating groups. See also Table S1. RP/HPLC-Q/TOF-MS/MS: Reversed-Phase High-Performance Liquid Chromatography-Quadrupole-Time of Flight Mass Spectrometry; HILIC-Q/TOF-MS/MS: Hydrophilic Interaction Liquid Chromatography–Quadrupole-Time of Flight Mass Spectrometry; PCA: Principal Component Analysis; PLS-DA: Partial Least Squares Discriminant Analysis; ROC: Receiver Operating Characteristic.

In a recent study, we analysed changes in faecal microbiota profiles associated with the initial histological alterations of the intestinal mucosa in the context of CRC [23]. The aim of the present work was to shed light on shifts in the faecal metabolome that could be associated with the histological changes occurring in the intestinal mucosa as a function of the type of morphological alteration (hyperplastic polyps and conventional adenomas) prior to the onset of CRC. The flowchart of the present study is summarized in Figure 1.

## 2. Results

### 2.1. General Characteristics of the Sample Population

A general description of the human sample population according to the main anthropometric characteristics, colonoscopy screening groups, and histopathological examination of mucosal biopsies is provided in Table 1. After colonoscopy and histopathological analyses, a total of 54 patients were classified into two main diagnosis groups: control (*n* = 20) and polyps (*n* = 34). Within the polyps group, 9 volunteers presented hyperplastic polyps and 25 displayed conventional adenomas. Males predominated in the polyps’ group, whereas females predominated in the control group.

### 2.2. Faecal Untargeted Metabolomics Profiles

Data processing and compound filtering (excluding pharmaceutical drugs) of faecal samples allowed for the tentative identification of 299 compounds in the 54 samples analysed (Table S1).

Comparison of groups two by two was carried out. In this way, controls and polyps were compared, as well as hyperplastic polyps and conventional adenomas. In order to determine the specific changes occurring in faecal samples from individuals bearing each type of polyps, the control group was separately compared with individuals displaying hyperplastic polyps or with conventional adenomas. MetaMapps were constructed for each of these comparisons to obtain metabolomics networks for the tentatively identified metabolites.

### 2.3. Control Group Versus Polyps Group

**Figure 2:**
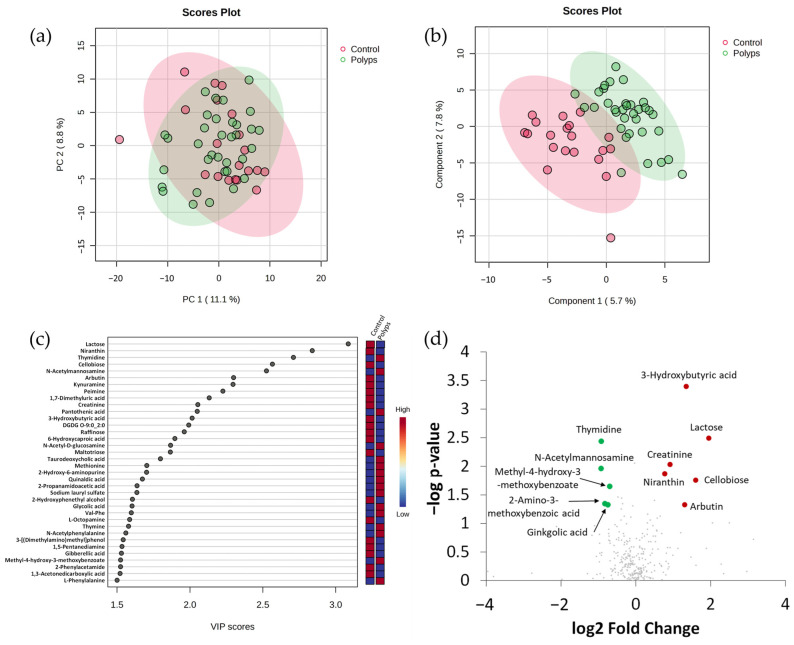
Faecal metabolome comparison between diagnosis groups (control and polyps) using integrated datasets from the two chromatographic platforms. (**a**) Principal Component Analysis (PCA) score plot. (**b**) Partial Least Squares Discriminant Analysis (PLS-DA) score plot. (**c**) Representation of metabolites with Variable Importance in Projection (VIP) scores > 1.5; red and blue colours for the different metabolites in vertical bars indicate the relative higher (red) or lower (blue) intensities in the comparison. (**d**) Volcano plot representation of significant metabolites obtained by U Mann–Whitney test (*p* < 0.05); red and green spots indicate, respectively, metabolites with a Fold Change (FC) > 1.5 or <0.6 in the comparison of control vs. polyps groups. Grey spots indicate metabolites not showing statistically significant differences between groups.

Evaluation by Principal Component Analysis (PCA) did not separate groups (Figure 2a), whereas the Partial Least Squares Discriminant Analysis (PLS-DA) showed a partial segregation of faecal samples from control and polyps (Figure 2b). The 36 most discriminating compounds with Variable Importance in Projection (VIP) scores > 1.5 are represented in Figure 2c. A total of 11 metabolites with Fold Change (FC) > 1.5 or <0.6 presented statistically differential abundances between groups by a Mann–Whitney U test (Figure 2d, Table S1). Six of these metabolites are from a probable exogenous origin. Five of them are compounds found in vegetables (cellobiose, arbutin, niranthin, ginkgolic acid and methyl-4-hydroxy-3-methoxybenzoate), whereas lactose is a component of milk and dairy products. Lactose, niranthin, arbutin, and cellobiose had significantly higher values in faeces from the control group, whereas the other two compounds were more abundant in faeces of individuals with intestinal polyps. Among the rest of the metabolites, one of them also had significantly higher values in faeces of the control group (creatinine, which is related with the general amino acids metabolism), whereas other three metabolites (thymidine and N-acetylmannosamine, which are related to pyrimidine and amino sugar metabolism, respectively, and 2-amino-3-methoxybenzoic acid, which is involved in the metabolism of tryptophan) presented more intensity in the polyps group. Finally, the ketone body 3-hydroxybutyrate was upregulated in the control group. These results indicate that several compounds from dietary exogenous sources contribute to differentiating stool samples of individuals belonging to the control group from those belonging to the group of intestinal polyps, whereas some other compounds, probably related with the endogenous metabolism of the host, also contribute to the differentiation.

### 2.4. Hyperplastic Polyps Versus Conventional Adenomas

**Figure 3:**
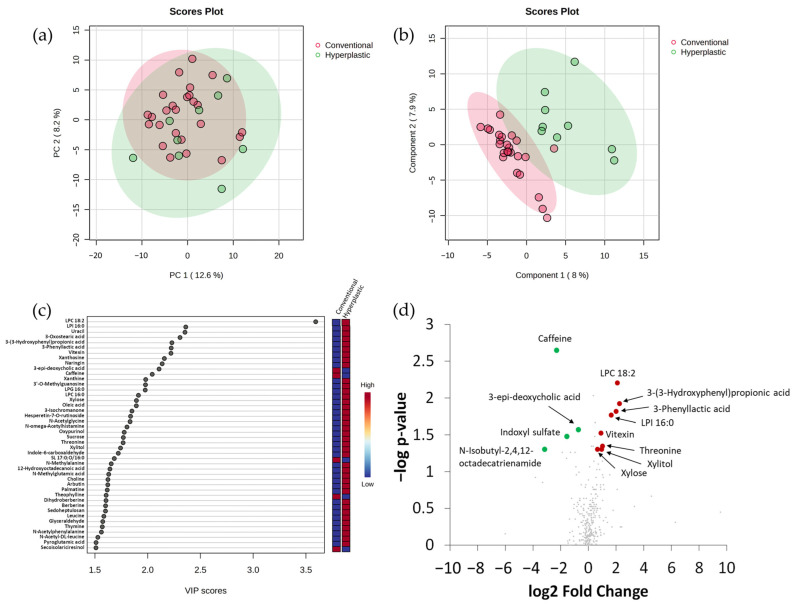
Faecal metabolome comparison between hyperplastic polyps and conventional adenomas using integrated datasets from the two chromatographic platforms. (**a**) Principal Component Analysis (PCA) score plot. (**b**) Partial Least Squares Discriminant Analysis (PLS-DA) score plot. (**c**) Representation of metabolites with Variable Importance in Projection (VIP) scores > 1.5; red and blue colours for the different metabolites in vertical bars indicate the relative higher (red) or lower (blue) intensities in the comparison. (**d**) Volcano plot representation of significant metabolites obtained by U Mann–Whitney test (*p* < 0.05); red and green spots indicate, respectively, metabolites with a Fold Change (FC) > 1.5 or <0.6 that were significant in the comparison of hyperplastic polyps vs. conventional adenomas groups. Grey spots indicate metabolites not showing statistically significant differences between groups.

A comparison was made between stools from the group of hyperplastic polyps and conventional adenomas in order to assess the main metabolomics features differentiating both types of lesions. PCA did not separate groups (Figure 3a), whilst PLS-DA provided an optimal separation for most samples between groups (Figure 3b). A total of 44 metabolites presented VIP scores > 1.5 (Figure 3c). Twelve compounds also displayed FC > 1.5 or <0.6 with *p*-value < 0.05 (Figure 3d; Table S1). Four of these metabolites were from a probable exogenous origin, mostly vegetable foods and derivatives, including vitexin (flavonoids), xylose, xylitol, and caffeine (a xanthine alkaloid). These metabolites, except for caffeine, had higher values in the group of hyperplastic polyps. 3-(3-hydroxyphenyl)propionic acid, with significantly higher values in samples from hyperplastic polyps, is a product derived from flavonoids [24,25]. Other significantly differential compounds include two glycerophospholipids (LPC 18:2 and LPI 16:0), a fatty acid amide (N-isobutyl-2,4,12-octadecatrienamide), 3-phenyllactic acid, threonine, and 3-epi-deoxycholic acid (bile acid), all of them but the bile acid and the fatty acid amide being more abundant in samples from hyperplastic polyps than in samples from the group of conventional adenomas. Finally, indoxyl sulfate, a tryptophan metabolite, was differentially more abundant in conventional adenomas. Our results indicate that metabolites derived from exogenous vegetable sources, some glycerophospholipids and fatty acid amides, bile acids, and metabolic features related to tryptophan metabolism mainly differentiate stool samples from individuals with hyperplastic polyps and conventional adenomas.

### 2.5. Controls Versus Hyperplastic Polyps

**Figure 4:**
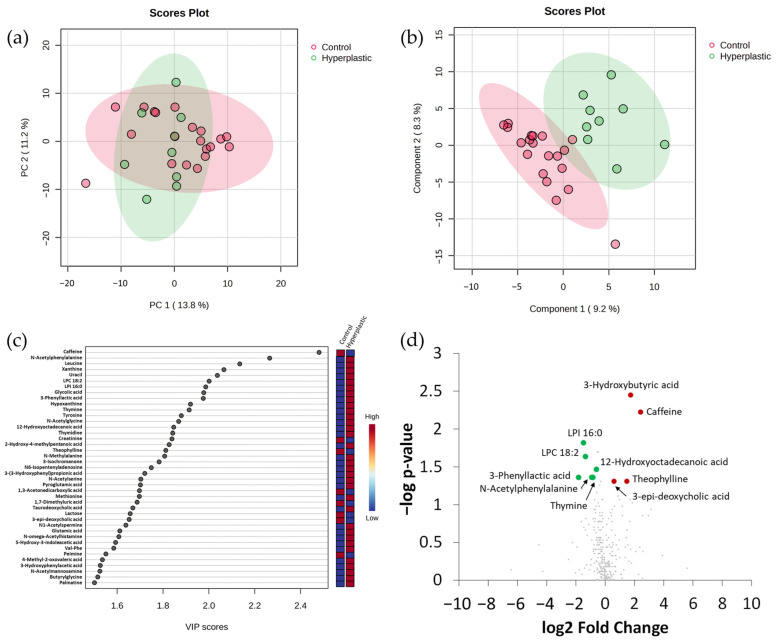
Faecal metabolome comparison between control group and hyperplastic polyps using integrated datasets from the two chromatographic platforms. (**a**) Principal Component Analysis score plot. (**b**) Partial Least Squares Discriminant Analysis (PLS-DA) score plot. (**c**) Representation of metabolites with Variable Importance in Projection (VIP) scores > 1.5; red and blue colours for the different metabolites in vertical bars indicate the relative higher (red) or lower (blue) intensities in the comparison. (**d**) Volcano plot representation of significant metabolites by U Mann–Whitney test (*p* < 0.05) with a Fold Change (FC) > 1.5 or <0.6; red and green spots indicate, respectively, metabolites with a Fold Change (FC) > 1.5 or <0.6 that were significant in the comparison of control vs. hyperplastic polyps’ groups. Grey spots indicate metabolites not showing statistically significant differences between groups.

To identify changes that specifically affected the faecal metabolome of individuals with hyperplastic polyps, we compared their metabolomes with those of the control group. No separation was obtained by PCA between groups (Figure 4a), whereas PLS-DA partially discriminated faeces from the control and hyperplastic polyp groups (Figure 4b). Forty-one compounds with VIP scores > 1.5 were the main contributors to the differences found (Figure 4c). Ten metabolites with FC > 1.5 or <0.6 displayed statistically significant differences in their values between control and hyperplastic samples (Figure 4d; Table S1). Caffeine and theophylline (xanthines) were the two compounds of exogenous origin displaying more intensity in samples from the control group. The other compounds found differentially more abundant in controls included 3-epi-deoxycholic acid and 3-hydroxybutyric acid. The remaining metabolites presented significantly higher values in faeces from individuals with hyperplastic polyps and included two metabolites from the phenylalanine metabolism (N-acetylphenylalanine and 3-phenyllactic acid), two glycerophospholipids (LPC 18:2 and LPI 16:0), a long-chain fatty acid (12-hydroxyoctadecanoic acid), and thymine (pyrimidine).

Five of the compounds mentioned just above also presented significant differences in intensity in the previous comparison between hyperplastic polyps and conventional adenomas: LPC 18:2, LPI 16:0, 3-phenyllactic acid, 3-epideoxycholic acid, and caffeine. Our results point to clear alterations of caffeine values and metabolic features related to xanthines/nucleotides, phenylalanine, bile acids, glycerophospholipids, and fatty acids as the main signatures differentiating the hyperplastic polyps group. The Area Under Curve (AUC) in the Receiver Operating Characteristic (ROC) curve presented values > 0.7 for all metabolites, with FC > 1.5 or <0.6 displaying significant differences between controls and hyperplastic polyps (Table S1), except for LPC 18:2, confirming their discriminating power.

### 2.6. Control Versus Conventional Adenomas

**Figure 5:**
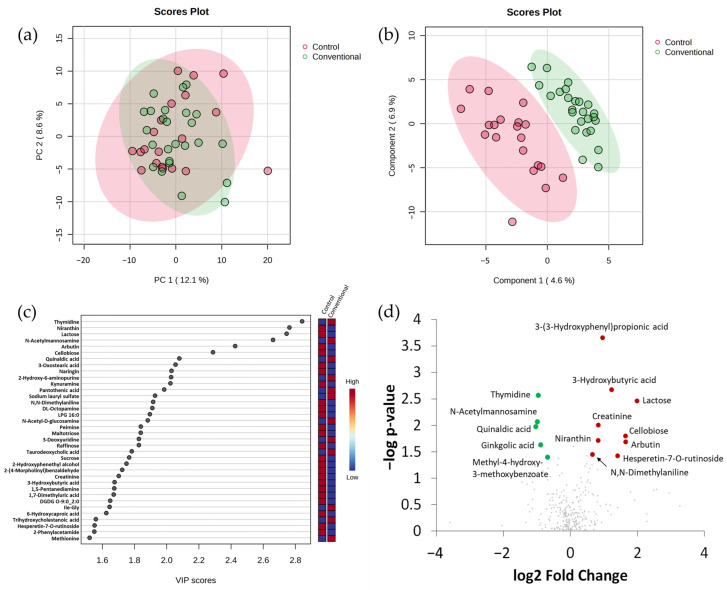
Faecal metabolome comparison between control group and conventional adenomas using integrated datasets from the two chromatographic platforms. (**a**) Principal Component Analysis score plot. (**b**) Partial Least Squares Discriminant Analysis (PLS-DA) score plot. (**c**) Representation of metabolites with Variable Importance in Projection (VIP) scores > 1.5; red and blue colours for the different metabolites in vertical bars indicate the relative higher (red) or lower (blue) intensities in the comparison. (**d**) Volcano plot representation of significant metabolites by U Mann–Whitney test (*p* < 0.05) with a Fold Change (FC) > 1.5 or <0.6; red and green spots indicate, respectively, metabolites with a Fold Change (FC) > 1.5 or <0.6 that were significant in the comparison of control vs. conventional adenomas groups. Grey spots indicate metabolites not showing statistically significant differences between groups.

The faecal metabolome of individuals diagnosed with conventional adenomas was compared with that of the control group in order to identify changes that affected specifically this type of polyps. The PCA did not separate groups (Figure 5a), whereas a complete separation between samples from the control group and conventional adenomas group was obtained with PLS-DA (Figure 5b). The 36 most discriminating compounds presenting VIP scores > 1.5 are represented in Figure 5c. Fourteen metabolites with FC > 1.5 or <0.6 displayed significant differences between groups (Figure 5d; Table S1). Remarkably, seven out of the nine metabolites presenting significantly higher values in the control group were from a probable exogenous dietary origin (lactose, cellobiose, arbutin, niranthin, hesperetin-7-O-rutinoside, N,N-dimethylaniline, and the flavonoids derivative 3-(3-hydroxyphenyl)propionic acid). 3-hydroxybutyric acid (ketone body) and creatinine (amino acids metabolism) were also differentially more abundant in the control group. The other five metabolites were found at significantly higher intensity in the group of conventional adenomas and were related to nucleotide/nucleoside metabolism (thymidine, and N-acetylmannosamine), tryptophan metabolism (quinaldic acid), or exogenous dietary compounds (ginkgolic acid and methyl-4-hydroxy-3-methoxybenzoate).

The values of one of the compounds mentioned just above, 3-(3-hydroxyphenyl)propionic acid, were also found to be significantly different in the comparison between hyperplastic polyps and conventional adenomas. The results obtained suggest that several metabolites from a probable dietary exogenous origin, together with compounds related with tryptophan and nucleotide/nucleosides metabolism, were the most relevant in characterizing the conventional adenomas group. From the fourteen metabolites with FC > 1.5 or <0.6 that were significant in the comparison between control and conventional adenomas, 10 of them presented an AUC value in ROC curves of > 0.7 (Table S1), which confirms the acceptable discriminating power for the following metabolites: 3-hydroxybutyric acid, lactose, thymidine, 3-(3-hydroxyphenyl)propionic acid, creatinine, N-acetylmannosamine, niranthin, cellobiose, arbutin, and quinaldic acid.

### 2.7. Global Metabolomics Analyses

**Figure 6:**
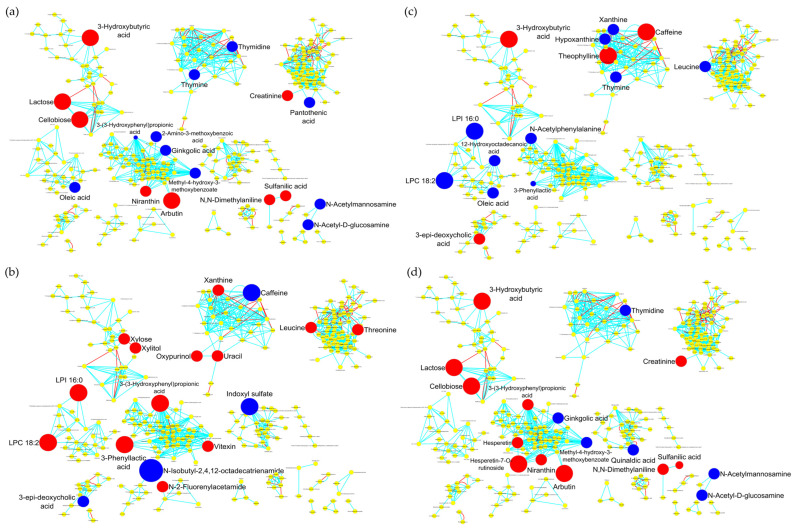
MetaMapp visualization of metabolomics data highlighting the differential metabolic regulation for the comparison. (**a**) Controls vs. polyps. (**b**) Hyperplastic polyps vs. conventional adenomas. (**c**) Controls vs. hyperplastic polyps. (**d**) Controls vs. conventional adenomas. Red edges denote KEGG reactant pair links and light blue edges symbolize Tanimoto chemical similarity at T > 700. Node sizes reflect fold change. Significantly increased metabolites with FC > 1.5 are in red colour, and metabolites significantly decreased with FC < 0.6 are represented in blue. Metabolites not significantly altered are represented as yellow nodes.

To globally visualize the metabolomics data and connections among metabolites, all compounds identified were mapped with the MetaMapp (2020) tool (Figure 6). Biochemical networks were constructed using the KEGG reactant pairs database for biochemical similarity and Tanimoto substructure composition matrices for chemical similarity, hence obtaining clusters of compounds representing larger modules. Overall, networks showed specific differences between polyps and the control group and between the two types of polyps (hyperplastic polyps and conventional adenomas). These differences were not randomly distributed but focused on specific types of compounds into given modules.

The biochemical representation of significant metabolomics changes between control and polyps evidenced that seven metabolic modules were affected (Figure 6a). Of them, one module including disaccharides (lactose, and cellobiose) plus the ketone body 3-hydroxybutyric acid and another module containing sulfanilic acid and N,N-dimethylaniline were overrepresented in faecal samples of the control group, whereas modules including pyrimidines (thymine and thymidine) and aminosugars (N-acetylmannosamine and N-acetyl-D-glucosamine) were underrepresented in controls. Differences between controls and polyps also included alterations in a module containing some vegetable compounds and derivatives (niranthin, arbutin, ginkgolic acid, 3-(3-hydroxyphenyl)propionic acid, and methyl-4-hydroxy-3-methoxybenzoate) together with a compound from the metabolism of tryptophan (2-amino-3-methoxybenzoic acid) and in another module related to the metabolism of amino acids (creatinine and pantothenic acid), as well as the underrepresentation of oleic acid in a separate module in samples from the control group.

The comparison of samples from hyperplastic polyps versus conventional adenomas pointed to clear differences in a module including compounds from the caffeine/xanthines/purines and pyrimidines metabolism (Figure 6b). Four relevant modules, including glycerophospholipids (LPI 16:0, LPC 18:2), monosaccharides (xylose and xylitol), amino acids (leucine, threonine), the flavonoid vitexin, 3-(3-hydroxyphenyl)propionic acid, and 3-phenyllactic acid, were all overrepresented in the group of hyperplastic polyps. On the other hand, the bile acid 3-epi-deoxycholic, the tryptophan metabolite indoxyl sulfate, and the fatty acid amide N-isobutyl-2,4,12-octadecatrienamide, all of them in separate modules, decreased in hyperplastic polyps versus conventional adenomas, whereas in the same comparison, the exogenous compound N-2-fluorenylacetamide was increased.

The comparison between the control group and hyperplastic polyps evidenced that five compounds in the module representing the caffeine/xanthines/purines and pyrimidines metabolism were altered with respect to controls (Figure 6c). Two other modules, one containing glycerophospholipids (LPI 16:0 and LPC 18:2) and another containing phenylalanine metabolites, as well as the amino acid leucine, were all underrepresented in the control group with respect to hyperplastic polyps, whereas the 3-epi-deoxycholic acid was overrepresented in the same comparison.

Finally, the comparison of samples from the control group versus conventional adenomas highlights the overrepresentation in controls of elements of exogenous origin distributed in two different modules, one containing four phenolic compounds (arbutin, niranthin, hesperetin, and herperetin-7-O-rutinoside) and another containing two disaccharides (lactose and cellobiose) plus 3-hydroxybutyric acid (Figure 6d). Sulfanilic acid/N,N-dimethylaniline (benzenesulfonic acids, exogenous compounds) and creatinine, in separate modules, were overrepresented in controls, whereas quinaldic acid (tryptophan metabolism), thymidine, and N-acetylmannosamine/N-acetyl-D-glucosamine were overrepresented in conventional adenomas.

Overall, these results are consistent with analyses comparing metabolites separately and demonstrate that some modules, including phenolic compounds and derivatives, di- and tri-saccharides, glycerophospholipids and fatty acids, caffeine/xanthines/purines and pyrimidines, bile acids, and amino acids and related compounds, were mainly altered in a way related to the histological lesions of the intestinal mucosa. However, whereas in conventional adenomas, the main differential signature was the underrepresentation of phenolic compounds and disaccharides and the alteration of the tryptophan metabolism with respect to controls, in hyperplastic polyps, the alterations in the metabolism of caffeine/xanthines/purines and pyrimidines and the overrepresentation of glycerophospholipids were the main differential characteristics.

## 3. Discussion

Metabolomics has been proposed as a non-invasive and promising method for the diagnosis and prognosis of CRC [26,27,28]. Metabolite alterations in different human samples, mainly serum, urine, and stools, have been reported in CRC, and some recent studies have focused on metabolomics alterations linked to histological lesions of the intestinal mucosa, before CRC onset [19,20,21,28,29,30]. In spite of this, comparatively few reports are still available on the metabolomics of the serrated pathway of carcinogenesis [31,32,33]. Therefore, in the present work, we have analysed changes in faecal metabolomics profiles associated with histological alterations of the intestinal mucosa according to clinical diagnosis groups (controls and polyps), as well as considering the type of polyps (hyperplastic and conventional adenomas), in order to establish specific faecal metabolic profiles of each type of alteration.

Metabolites found to be different between the control and polyps groups mainly included some phenolic compounds; disaccharides; compounds from the metabolism of xanthines/purines and pyrimidines and from the metabolism of amino acids; as well as ketone bodies, amino sugars, and methoxybenzoic acids. Alterations of some of these metabolites and their corresponding metabolic pathways have previously been reported in CRC and in individuals with conventional adenomas using faeces or other biological samples for analyses [13,16,19,20,21,30,31,32,34]. Compounds of exogenous origin that were overrepresented in control samples with respect to those from the polyps group included niranthin (phenolic compound: lignan) and arbutin (phenolic glycoside), which are components of some fruits and vegetables [35,36]. These and other phenolic compounds, including vitexin, hesperetin-7-O-rutinoside (hesperidin) and its aglycone hesperetin, which were also differential in some comparisons of the present work, are considered diet-derived bioactive compounds with antioxidant and anti-inflammatory properties, as well as possible anticarcinogenic effects [35,37,38,39,40]. Cellobiose is a disaccharide found in some dietary vegetables, whereas lactose is mainly provided in the diet by milk and dairy products. Possible explanations for the higher intensity of these compounds in samples from the control group may be related to differences in dietary patterns, intestinal absorption, the interactions of these compounds with the intestinal microbiota, or alterations of the host endogenous metabolism in the polyps group. Methyl-4-hydroxy-3-methoxybenzoate (methyl vanillate) and 2-amino-3-methoxybenzoate (3-methoxyanthranilate) are methoxybenzoic acids overrepresented in the group of polyps, being the first compound to also be overrepresented in conventional adenomas as compared with controls. Methyl-4-hydroxy-3-methoxybenzoate could have an exogenous dietary origin or can be formed endogenously as a by-product of caffeic acid or as a metabolite present in some foods from vegetable origin, whereas the second one is a metabolite from the catabolism of tryptophan through the kynurenine pathway. Differences found in our work for these two compounds could reflect differential intake of caffeic acid and tryptophan or alterations in the metabolism of these two precursors. In this respect, a dysregulation of tryptophan metabolism has been linked to colorectal tumorigenesis [41], and elevated levels of circulating tryptophan–kynurenine pathway metabolites were associated with worse prognosis in patients with CRC [42].

Among compounds that can originate from the host endogenous metabolism, 3-hydroxybutyric acid has been found at higher intensity in faecal samples of individuals in the control group. 3-hydroxybutyric acid is a partial degradation product formed in the liver from branched-chain amino acids released from the muscle, or it could be also generated by oxidization of lipids from the adipose tissue. This compound acts as a fuel for certain tissues in ketosis, also being a precursor of acetoacetyl-CoA and acetyl-CoA for the synthesis of cholesterol and complex lipids [43]. Some beneficial health effects have been attributed to 3-hydroxybutyric acid, such as the reduction in the proliferation of colonic crypt cells and potential suppression of tumour growth [44]. Remarkably, this compound has been found to be increased in some metabolic adverse conditions such as adenocarcinoma or adenomatous intestinal polyps [28,29,45,46]. A differential role could be attributed to 3-hydroxybutyric acid based on the so called “3-hydroxybutyrate paradox”, by which, at low concentrations and in the absence of a Warburg effect, this compound could act as an oxidative energy source, whereas at high concentrations it could mediate histone acetylation, contributing to inhibit cell proliferation [47]. The significantly different levels of 3-hydroxybutyric acid in samples from the control and polyps groups may suggest a different action of 3-hydroxybutyric acid in each of the groups, as well as an altered metabolism of lipids and/or amino acids in the polyps group with respect to controls. Thymidine is a DNA pyrimidine nucleoside which is part of the DNA structure of all eukaryotic and prokaryotic cells. An enriched pyrimidine metabolism has been reported in faecal samples from patients with advanced adenomas and CRC, as well as in the tumour-associated metabolome of patients with high-grade dysplastic polyps [48]. The higher intensity of thymidine in the polyps group with respect to controls found in the present work could be related to cellular DNA alterations of the intestinal mucosa. Creatinine is a catabolic end-product from the amino acids arginine and glycine that is present in blood and is excreted by urine. This compound is used as an indicator of muscle and kidney function. Coincident with our results in the group of polyps, creatinine has been generally reported to be downregulated in different biospecimens of individuals with CRC, which reflects an alteration in the metabolism of proteins/amino acids and/or the kidney function [29,30].

N-acetylmannosamine is an acylaminosugar participating in several enzymatic reactions. This compound is a precursor of sialic acids that are part of glycoproteins and glycolipids as O-glycans and glycosaminoglycans, which have structural functions in several tissues of vertebrates. A deregulation of glycosylation occurs during the malignant transformation of cells, and components of glycosaminoglycans have been found to be upregulated in tumour tissues during the progression of CRC [29,49]. In agreement with these reports, we have found N-acetylmannosamine to be upregulated in faecal samples from the polyp group as compared to controls.

When comparing hyperplastic polyps and conventional adenomas, the main differences found in the faecal metabolome affected several lipids, mainly glycerophospholipids derived from choline (LPC 18:2) and inositol (LPI 16:0). Some phenolic compounds such as flavonoid glycosides, a monosaccharide (xylose), as well as caffeine and compounds related to the metabolism of xanthines/purines and pyrimidines and to the metabolism of phenylalanine and tryptophan were also affected. When compared with control group, more compounds were found to be significantly altered in conventional adenomas than in hyperplastic polyps. These results were in good agreement with the complete separation obtained in the PLS-DA analysis for conventional adenomas with respect to the control group and with the differential intestinal microbiota profiles recently reported for these two groups in the same sample population [23].

The significantly lower intensity of several phenolic compounds in faecal samples was a differential feature of the adenomas group when compared with hyperplastic polyps (vitexin) and controls (niranthin, arbutin, and hesperetin-7-O-rutinoside). Flavonoids such as vitexin, naringin, and hesperetin-7-O-rutinoside can be transformed to their corresponding aglycones by hydrolases from the intestinal epithelial cells or from the gut microbiota [50]. Moreover, other enzymatic activities of the microbiota can degrade flavonoid aglycones into small molecules in the gut [38,50,51,52]. Part of these metabolites can enter into the intestinal epithelial cells to be inactivated by phase II glucuronidases, methylases, and sulfatases, and then be pumped out by efflux transporters to bile or gut lumen [50]. Little is still known about the metabolism of phenolic compounds by the gut microbiota and its contribution to the general metabolism of phenol glycosides in comparison to the endogenous host metabolism. The lower values for 3-3-(hydroxyphenyl)propionic acid and 3-phenyllactic acid, two compounds produced by the activity of the gut microbiota, in the adenomas group vs. the group of hyperplastic polyps and vs. the control group (for 3-(3-hydroxyphenyl) propionic acid) could suggest a more limited microbial contribution to the metabolism of phenolic compounds by the microbiota of the adenomas group [53,54]. In this regard, we previously reported a different microbiota composition in individuals with conventional adenomas with respect to the group of hyperplastic polyps and controls [23]. The higher enrichment of quinaldic acid and indoxyl sulfate in faecal samples of individuals diagnosed with conventional adenomas vs. controls and hyperplastic polyps, respectively, points to an alteration in the route of degradation of tryptophan in conventional adenomas that seems not to occur in hyperplastic polyps. Quinaldic acid is formed from tryptophan through the kynurenine pathway and has anti-proliferative activity on cancer cells [55]. Kynurenine derivatives play a role in the attenuation of inflammatory responses and in the reduction in free radical formation in different parts of the body, with special relevance in the central nervous system [56,57]. Moreover, indoxyl sulfate is a uremic toxin formed by the combined action of intestinal bacteria over dietary tryptophan and the subsequent transformation of indole in the liver by cytochrome P450 enzymes [58].

Significant lower intensity of caffeine and theophylline, together with higher values of thymine, was a differential signature of samples from hyperplastic polyps with respect to conventional adenomas and controls. This, together with the alterations found for some compounds in the metabolic module, including caffeine/xanthines/purines and pyrimidines, suggests an enhanced degradation of caffeine and alterations in the metabolism of xanthines/purines and pyrimidines in the group of hyperplastic polyps with respect to the groups of conventional adenomas and controls. Caffeine is metabolized in the body by the cytochrome P450 enzyme CYP1A2, which is also involved in the phase I metabolism of many drugs and toxic compounds [59]. It has been indicated that high in vivo CYP1A2 activity could be related with a higher cancer risk on the basis that exposure to xenobiotics increases the activity of this enzyme and because some xenobiotics have been found to be involved in the aetiology of several types of cancers [60,61]. However, a low activity of CYP1A2 has also been linked to CRC [62]. Some studies point to the enrichment of caffeine metabolism and to alterations in purine and pyrimidine metabolism as well as in the xanthine/hypoxanthine ratio in samples of individuals with polyps and CRC [32,35,47,48,63,64]. However, there are no previous metabolomics comparisons between the serrated and the adenomatous pathways of carcinogenesis. Modulation of the gut microbiota by coffee consumption, one of the main dietary sources of caffeine, has been documented in several studies, and it has been shown that several bacteria from different genera are able to metabolize caffeine [65,66,67]. However, it is not clear at present whether caffeine could be actively metabolized by the intestinal microbiota. Because of that, metabolomics changes evidenced in our work for caffeine could be attributed preferentially to alterations in the endogenous metabolism of the host. Nevertheless, the higher levels in the group of hyperplastic polyps of glycolic acid, a metabolite that can be produced by some yeasts and bacteria, as compared to controls prompts us not to dismiss a hypothetical contribution of the intestinal microbiota to the altered levels of caffeine [68,69].

The higher intensity in faeces of some glycerophospholipids derived from inositol and choline differentiated the group of hyperplastic polyps from that of conventional adenomas; in addition, two of these compounds were also differential in the comparison of hyperplastic polyps with controls. The enrichment of phospholipids and glycerophospholipids has been repeatedly reported in CRC and high-risk polyps, explained by the higher metabolic turnover and enhanced membrane biosynthesis associated with cell proliferation [28,29,30,32,37,48,70,71]. Notably, as intestinal mucosa lesions of the individuals participating in the present study have not reached a malignant or pre-malignant cell status, the differentially higher intensity of glycerophospholipids in samples from individuals with hyperplastic polyps represents a specific differential signature of these type of lesions.

Interestingly, a comparatively higher presence of the potentially carcinogenic dye N,N-dimethylaniline was found in faecal samples from controls versus conventional adenomas. This compound is detoxified in the liver by the action of a flavin-monooxygenase [72]; the differences found could be due to differential exposure or to a more activated detoxification mechanism in individuals with conventional adenomas.

Our statistical analyses, followed by ROC curves for individual metabolites, confirmed the potential discriminatory power for some of them. Further studies in a wider population would allow us to confirm the combinations of some of these metabolites as potential biomarkers for discriminating groups of intestinal mucosa lesions in the context of CRC. In this respect, two recent studies analysing plasma and faecal metabolomes in wider populations found suitable markers of the adenoma–carcinoma progression, evidencing a better discriminating power for plasma than for faecal metabolites [19,21].

In the research for early biomarkers of CRC, the potential of mi-RNA should be also explored [73]. Considering the influence and the interrelationship existing among microbiota, metabolism, and the immune system in the context of CRC, faecal microbiota transplantation could become as a cornerstone in CRC treatment and prevention [74].

This study has several limitations. First, the low sample size limits the power of the study. Second, we have described some metabolites from a probable exogenous origin (diet) that have higher intensity in controls than in individuals with polyps; hence, human dietary intervention studies should be considered in the future targeting these and other exogenous metabolites. Third, our study was focused on a specific geographic region in the North of Spain; therefore, large-scale or multi-centre investigations are desirable in order to generalize our observations. Fourth, the low sample size and the geographical limit in recruiting volunteers makes the proposal of sound biomarker candidates for the progression of intestinal mucosal lesions difficult. Despite these limitations, our findings provide meaningful insights into the significance of faecal metabolome shifts in the progression of different types of intestinal mucosa lesions prior to CRC development.

## 4. Materials and Methods

### 4.1. Study Design and Volunteers

The recruitment was carried out as described by Ruiz-Saavedra et al. [75]. Volunteers were randomly recruited by trained physicians from among those subjects of the Digestive Section at the Central University Hospital of Asturias (HUCA) or the Carmen and Severo Ochoa Hospital in Asturias (North Spain) for consultation about clinical symptoms or as part of the CRC screening program in the region between October 2019 and December 2021. To enrol in the study, the inclusion criteria were: volunteers aged between 40 and 79 years that were submitted to a screening colonoscopy per protocol. The following exclusion criteria were applied: previous surgery of the digestive system, immune-related disease, treatment with medical drugs including antibiotics, and having specific cancer treatment at the time of the study or in the previous two months. Volunteers were first informed about the objectives and procedures of the study and signed informed consent forms. Anamnesis was performed and faecal samples were collected at the time of recruitment prior to preparation of patients for colonoscopy. Biopsies of intestinal mucosa and polyps were resected during colonoscopy and examined at the Department of Anatomical Pathology of HUCA, as described elsewhere [75]. Individuals were classified according to the colonoscopy and histopathological examination. The group of “controls” included individuals with non-pathological results for colonoscopy, confirmed by the absence of mucosal lesions by histopathological analysis. The group of “polyps” included those individuals with intestinal alterations detected through colonoscopy and confirmed by histopathological analyses. This group included individuals with either hyperplastic polyps or conventional adenomas, confirmed by morphological features detected by histopathological analysis. The present study has been evaluated and approved by the Regional Ethics Committee of Clinical Research of Asturias (Ref. 163/19) and the Committee on Bioethics of CSIC (Ref. 174/2020). The study was carried out following the fundamental principles of the Declaration of Helsinki, the Council of Europe Convention on Human Rights and Biomedicine, and the Spanish legislation on bioethics. Personal data protection was treated according to the Directive 95/46/EC of the European Parliament and the Council of October 1995 on the protection of individuals.

### 4.2. Untargeted Metabolomic Analyses

#### 4.2.1. Chemicals and Reagents

LC–MS-grade acetonitrile (ACN) and methanol were obtained from VWR Chemicals (Barcelona, Spain), whereas ultrapure water was obtained from a Millipore system (Billerica, MA, USA). Formic acid was purchased from Fisher Scientific (Waltham, MA, USA).

#### 4.2.2. Metabolite Extraction

Stool samples were stored after collection in aliquots at −20 °C and thawed on ice prior processing. The weighted and dried samples (100 mg) were mixed with 1 mL of 80% (*v*/*v*) methanol at −20 °C and mixed for 10 min at 4 °C and 2000 rpm. Then, ultrasound was applied for 30 min at room temperature. Finally, samples were centrifuged at 14,800 rpm for 15 min at 4 °C, and the supernatant was collected, divided in two fractions, and stored at −80 °C until chromatographic analyses.

#### 4.2.3. Reversed-Phase High-Performance Liquid Chromatography–Quadrupole-Time of Flight Mass Spectrometry (RP/HPLC-Q/TOF-MS/MS) Analysis

Aliquots of 2 μL from the first fraction were directly injected (three technical replicates) into a HPLC model 1290 (Agilent Technologies, Waldbronn, Germany), and compounds were separated using a Zorbax Eclipse Plus C18 analytical column (100 mm × 2.1 mm id; 1.8 μm particle size) equipped with an Zorbax C18 pre-column (5 mm × 2.1 mm id, 1.8 µm), both from Agilent Technologies (Wadbronn, Germany). The column temperature was held at 40 °C and the flow rate was set at 0.5 mL/min. Milli-Q water was used as mobile phase (A), acetonitrile (ACN) as mobile phase (B), and 0.1% formic acid as a mobile phase modifier. The gradient started at 0 min with 0% (B), 0–30% (B) for 7 min, 30–80% (B) for 2 min, 80–100% (B) for 2 min, 100% (B) for 2 min, and 3 min of post-time to return to initial conditions. Compounds were eluted into a Q/TOF series 6540 from Agilent Technologies (Waldbronn, Germany) equipped with an Agilent Jet Stream thermal orthogonal ESI source. The mass spectrometer was operated in ESI positive mode using the following parameters: capillary voltage, 3000 V; mass range, 25–1100 *m*/*z*; nebulizer pressure, 40 psig; drying gas flow rate, 8 L/min; dry gas temperature, 300 °C. The sheath gas flow was 11 L/min at 350 °C. Method blanks and a pooled mixture of all samples were included as quality control samples and subjected to iterative MS/MS with mass error tolerance of 20 ppm and retention time (RT) exclusion tolerance of ±0.2 min to increase the coverage of the MS/MS spectra acquired. MS/MS spectra were acquired employing the auto MS/MS mode using 5 precursors per cycle, dynamic exclusion after two spectra (released after 0.5 min), and collision energies of 20 and 40 V. Mass accuracy was corrected using ions at *m*/*z* 121.0509 (C_5_H_4_N_4_) and 922.0098 (C_18_H_18_O_6_N_3_P_3_F_24_), which were simultaneously pumped into the ion source.

#### 4.2.4. Hydrophilic Interaction Liquid Chromatography–Quadrupole-Time of Flight Mass Spectrometry (HILIC-Q/TOF-MS/MS) Analysis

A sample of 50 μL of the second fraction (three technical replicates) was evaporated and resuspended in 40 μL of ACN:water (80:20, *v*/*v*) with a mixture of internal standard compounds (CUDA, DL-alanine-d3, DL-glutamic acid-d3, 15N2-L-arginine, L-methionine-d8 and Val-Tyr-Val). Aliquots of 5 μL were then injected into the same instrument as specified above, and compounds were separated using an Acquity UPLC BEH Amide column (150 mm × 2.1 mm id; 1.7 μm particle size) equipped with an Acquity UPLC BEH Amide VanGuard Pre-column (5 mm × 2.1 mm id, 1.7 µm), both from Waters (Eschborn, Germany). The column temperature was held at 45 °C and the flow rate was set at 0.4 mL/min. Milli-Q water was used as mobile phase (A), 95:5 (*v*/*v*) ACN:water was used as mobile phase (B), and 10 mM ammonium formate and 0.125% formic acid were used as a mobile phase modifiers. The gradient started with 2 min at 100% (B), 100–70% (B) for 5.7 min, 70–40% (B) for 1.8 min, 40–30% (B) for 0.75 min, 30–100% (B) for 2.5 min, and 4 min at 100% (B) to return to initial conditions. Compounds were eluted into the same mass spectrometer as described before. The mass spectrometer was operated in ESI negative mode using the following parameters: capillary voltage, −3500 V; mass range, 50–1700 *m*/*z*; nebulizer pressure, 35 psig; drying gas flow rate, 11 L/min; dry gas temperature, 200 °C. The sheath gas flow was 11 L/min at 350 °C. Method blanks and a pooled mixture of all samples were included as quality control samples and were subjected to iterative MS/MS using the same parameters as described before. Mass accuracy was corrected using ions at *m*/*z* 119.0363 (C_5_H_4_N_4_) and 966.0007 (C_18_H_18_O_6_N_3_P_3_F_24_) simultaneously pumped into the ion source.

#### 4.2.5. Data Processing

Data obtained from each analytical platform (RP/HPLC-Q/TOF-MS/MS and HILIC-Q/TOF-MS/MS) were converted to ABF format and processed separately by MS-DIAL software (v. 4.8). In-house RT-*m/z* libraries, the public LipidBlast MS/MS spectral library, and the NIST20 MS/MS database were used for metabolite annotation. For RP/HPLC-Q/TOF-MS/MS data, the parameters used were the same as described elsewhere [76]. For HILIC-Q/TOF-MS/MS data, the parameters used were: retention time, 0–17 min; mass range, 50–1700 Da; MS1 tolerance, 0.01 Da; MS2 tolerance, 0.025 Da; minimum peak height, 1000; accurate mass tolerance (MS1 and MS2) for MSP library, 0.01 Da and 0.025 Da; identification score cut off for MSP library, 80%; retention time tolerance for RT-*m/z* library, 0.1 min; accurate mass tolerance for RT-*m/z* library, 0.01 Da; identification score cut off for RT-*m/z* library, 85%. CUDA, DL-alanine-d_3_, DL-glutamic acid-d_3_, 15N_2_-L-arginine, L-methionine-d_8_, and Val-Tyr-Val internal standard compounds were used for retention time correction. Metabolites were annotated following the Metabolomics Standard Initiative (MSI) guidelines [77]: MSI level 1 for metabolites with precursor *m/z*, in-house RT-*m/z* libraries, and MS/MS spectral library matching; MSI level 2a for metabolites with precursor *m/z* and MS/MS spectral library matching, and MSI level 2b for metabolites with precursor *m/z* and in-house RT-*m/z* library matching. The list of metabolites obtained from each analytical technique was filtered by removing unknown metabolites, metabolites with a maximum peak height below three times the average height in the method blanks, and metabolites with a maximum height below 1000 units. Missing values were imputed by half of the minimum height value, the median of the three technical replicates was calculated, and the data were processed using the bioinformatic tool MS-FLO (https://msflo.fiehnlab.ucdavis.edu/, accessed on 2 January 2024). Duplicated metabolites and isotopes were removed, the heights of the different adducts from the same compound were combined, and Systematic Error Removal using the Random Forest normalization method was applied for each data set using the pool mixtures as reference samples [78]. In the case of compounds identified by the two analytical platforms, only those presenting the lower relative standard deviation in the pool mixtures were retained. Moreover, those metabolites identified as pharmaceutical drugs were excluded from further statistical analyses.

The InChiKey or compound names were imported into the web-based Chemical Translation Service (https://cts.fiehnlab.ucdavis.edu/batch, accessed on 2 January 2024) to obtain the PubChem Compound Identifiers (CID). The Kyoto Encyclopedia of Genes and Genomes identifiers (KEGG ID) and the Human Metabolome Database identifiers (HMDB ID) were obtained using the Compound ID Conversion module from MetaboAnalyst 6.0 web-based software (https://www.metaboanalyst.ca/MetaboAnalyst/upload/ConvertView.xhtml, accessed on 2 January 2024). Simplified molecular-input line-entry system (SMILES) codes were obtained from the PubChem Compound Identifier Exchange service (https://pubchem.ncbi.nlm.nih.gov/idexchange/idexchange.cgi, accessed on 2 January 2024). By using the bioinformatics tool MetaMapp (http://metamapp.fiehnlab.ucdavis.edu/ocpu/library/MetaMapp2020/www/, accessed on 2 January 2024), KEGG reactant pairs and Tanimoto similarity scores were obtained for the identified metabolites. Thereafter, Cytoscape software (v.3.7.2) was used to construct metabolic networks of chemical and biochemical reactions [76,79].

### 4.3. Statistical Analysis

IBM SPSS software (version 25.0; IBM SPSS, Inc., Chicago, IL, USA) was used to analyse anthropometrical, clinical, and histopathological data. The categorical variables are presented as numbers and percentages, and continuous variables are presented as mean ± standard deviation. The Mann–Whitney U test was performed for pairwise comparison of continuous variables (*p* < 0.05), whereas the categorical variable “gender” was analysed by a chi-square test.

The integrated metabolomics dataset from the two chromatographic platforms was analysed using MetaboAnalyst 6.0 web-based software and the following two by two comparisons were performed: control vs. polyps, hyperplastic polyps vs. conventional adenomas, control vs. hyperplastic polyps, and control vs. conventional adenomas. PCA, followed by PLS-DA, was carried out considering VIP scores as significant when VIP > 1.5. Significant differences of metabolites between groups were also evaluated by the non-parametric Mann–Whitney U test and considered significant at *p* value < 0.05. Fold changes (FC) between groups were calculated for significant metabolites, and only those with FC > 1.5 or FC < 0.6 were arbitrarily considered. The AUC of univariate ROC curves were calculated using the “Biomarker Analysis” module and were considered acceptable to determine the diagnostic effectiveness of the metabolites when >0.7.

## 5. Conclusions

Hyperplastic polyps and conventional adenomas displayed altered metabolites in three main biochemical modules. The faecal metabolome of patients with hyperplastic polyps was enriched in glycerophospholipids and presented alterations in the degradation pathways of xanthines/purines and pyrimidines. In contrast, with respect to controls, the lower intensity in some phenolic compounds and disaccharides, all of them from a probable dietary origin, was the main characteristic in the faecal metabolomes of individuals with conventional adenomas. Further research should include integrated omics techniques and dietary assessment to elucidate the relationship among diet, microbiota, and metabolome during the initial histological changes in the intestinal mucosa in the context of CRC.

## Figures and Tables

**Table 1:** General characteristics of the sample population according to colonoscopy diagnosis and histopathological analysis of samples resected from the intestinal mucosa during colonoscopy.

Variables	Controls (*n* = 20)	Polyps (*n* = 34)
Female	14 (70.0%)	15 (44.1%)
Age (years)	60 ± 9	61 ± 6
BMI (kg/m^2^) *	25.67 ± 3.81	27.87 ± 4.16
Histopathological analysis:		
Non-pathological controls	20 (100%)	-
Hyperplastic polyps	-	9 (26.5%)
Conventional adenomas	-	25 (73.5%)

Values are presented as n (% in the sample population) or mean ± standard deviation. No significant differences (*p* ≥ 0.05) were found for gender (chi-square test), age, or BMI (Mann–Whitney U test) between diagnosis groups. (*) BMI calculations were determined in volunteers with available information (controls: 14, polyps: 27). Samples from individuals with more than one type of resected polyp were considered in the histopathological group of that of the higher risk. BMI: Body mass index.

## Data Availability

The original contributions presented in the study are included in the article/Supplementary Materials, further inquiries can be directed to the corresponding authors.

## Supplementary Materials

The following supporting information can be downloaded at: https://www.mdpi.com/article/10.3390/ijms252413324/s1.
